# Pad, psychiatry and stigma

**DOI:** 10.1192/j.eurpsy.2021.114

**Published:** 2021-08-13

**Authors:** E. Kowalinski, C. Huber

**Affiliations:** Psychiatry, University of Basel, Basel, Switzerland

**Keywords:** physician-assisted dying, Mental illness, suffering, terminality

## Abstract

In 2018, the Swiss Academy of Medical Sciences (SAMW) published a new guideline on physician-assisted dying (PAD). In line with the SAMW guideline published in 2004, the patients’ ability to judge, their self-determination, careful consideration and permanence of their wish to die as well as the lack of therapeutic options were set as necessary conditions. However, while the previous wording considered assisted suicide to be ethically justifiable if the patient’s condition is terminal, the new guideline requires that it is unbearable. This difference has been the subject of intense discussion in Swiss healthcare professionals and the population alike. This controversy is particularly important for those affected by mental illness who have a persistent desire to die. This is because mental disorders cannot usually be classified as terminal illnesses, but they can certainly lead to suffering that is perceived as unbearable. Furthermore, it is known that persons with mental illness are subject to stigmatization. It is therefore likely that there is a connection between the stigmatization of mentally ill people and the position on PAD for this group. This talk provides theoretical background on this discusion and proposes a study protocol to investigate the acceptance of PAD in relation to the type of illness as well as the factors of unbearable suffering and terminality. It will furthermore look into the criteria of the 2004 and 2018 guidelines and will explore if there is a connection between stigmatization and the assessment of whether a person should be granted access to assisted suicide.

**Disclosure:**

No significant relationships.

